# Recent Trends in the Incidence of Anxiety Diagnoses and Symptoms in Primary Care

**DOI:** 10.1371/journal.pone.0041670

**Published:** 2012-08-03

**Authors:** Kate Walters, Greta Rait, Mark Griffin, Marta Buszewicz, Irwin Nazareth

**Affiliations:** Department of Primary Care and Population Health, University College London (UCL), London, United Kingdom; Linkoping University, Sweden

## Abstract

**Background:**

Anxiety is common, with significant morbidity, but little is known about presentations and recording of anxiety diagnoses and symptoms in primary care. This study aimed to determine trends in incidence and socio-demographic variation in General Practitioner (GP) recorded diagnoses of anxiety, mixed anxiety/depression, panic and anxiety symptoms.

**Methodology/Principal Findings:**

Annual incidence rates of anxiety diagnoses and symptoms were calculated from 361 UK general practices contributing to The Health Improvement Network (THIN) database between 1998 and 2008, adjusted for year of diagnosis, gender, age, and deprivation. Incidence of GP recorded anxiety diagnosis fell from 7.9 to 4.9/1000PYAR from 1998 to 2008, while incidence of anxiety symptoms rose from 3.9 to 5.8/1000PYAR. Incidence of mixed anxiety/depression fell from 4.0 to 2.2/1000PYAR, and incidence of panic disorder fell from 0.9/1000PYAR in 1998 to 0.5/1000PYAR in 2008. All these entries were approximately twice as common in women and more common in deprived areas. GP-recorded anxiety diagnoses, symptoms and mixed anxiety/depression were commonest aged 45–64 years, whilst panic disorder/attacks were more common in those 16–44 years. GPs predominately use broad non-specific codes to record anxiety problems in the UK.

**Conclusions/Significance:**

GP recording of anxiety diagnoses has fallen whilst recording of anxiety symptoms has increased over time. The incidence of GP recorded diagnoses of anxiety diagnoses was lower than in screened populations in primary care. The reasons for this apparent under-recording and whether it represents under-detection in those being seen, a reluctance to report anxiety to their GP, or a reluctance amongst GPs to label people with anxiety requires investigation.

## Introduction

Anxiety disorders are very common in the community and yet there has been relatively little research focusing on them in comparison to other disorders, such as depression. Lifetime community prevalence rates of anxiety disorders reach between 16.6% and 28.8% [1], [2] worldwide, whilst in the Adult Psychiatric Morbidity Survey in England 2007 the two-week community prevalence was 9% for mixed anxiety and depression, 4.4% for Generalised Anxiety Disorder (GAD) and 1.1% for Panic Disorder [3]. In an international study in primary care populations, 5–10% of attenders screened using the Patient Health Questionnaire (PHQ) in general practice had an ‘other anxiety syndrome’ and a further 6–9% had ‘panic syndrome’ [4]. Anxiety disorders are associated with significant morbidity including distress, reduced quality of life and functional impairment [5], [6]. They are associated with significantly increased health care utilization [5] and increased costs, estimated at $42 billion dollars in direct and indirect costs in USA in the 1990s [7] and estimated at £14.2 billion in England in 2007, mostly through lost employment [8].

Very few studies have determined the incidence of anxiety disorders and anxiety symptoms, or explored trends in incidence over time. A systematic review of incidence and prevalence studies of anxiety disorders found an insufficient number of incidence studies available for inclusion to perform a meta-analysis, and concluded that further knowledge is required about the onset of anxiety disorders and in particular any social variables that may mediate the expression of these disorders [2]. More recent community survey data from the United States found an incidence of first ever episode of anxiety disorder of 1.58/100 person years, with increased incidence crudely associated with female gender, younger age and low income [9].

A number of studies have reported an under-detection of psychological disorders in general by General Practitioners (GPs) [6], [9]–[11]. One study based on GP recorded anxiety diagnoses in 2002–4 found a prevalence of 7.2% for all recorded anxiety disorders and symptoms combined [12], suggesting an under-detection or under-recording of anxiety in comparison to populations screened in GP waiting-rooms [4]. Previous work on depression has shown that in the last decade GPs have increasingly used symptoms rather than diagnostic labels to categorise their patients’ illnesses in their records [13]. We do not currently understand how GPs choose to define people presenting with anxiety symptoms and disorders, or how this may vary by socio-demographic characteristics and over time. Socio demographic factors such as gender and deprivation have been shown to have an effect on the incidence of other mental health disorders such as depression but much less is known about anxiety. The aim of this study was to describe socio-demographic trends in the recording of diagnoses and symptoms relating to anxiety, panic disorder and mixed anxiety and depression in primary care during the period 1998–2008.

## Methods

### Ethics Statement

The study was given a favourable opinion by the London Medical Research Ethics Committee (07/MRE02/5).

### Study Population & Setting

We used data from all adults aged 16 years and over registered with 361 general practices in the United Kingdom (UK) providing data to ‘The Health Improvement Network’ (THIN) during the period Jan 1998– Mar 2008. All participants had a minimum of one year follow-up data that met pre-defined quality standards.

### Data Source

The Health Improvement Network (THIN) database is a widely used primary care clinical longitudinal database with over 20 years of data on more than 6 million patients (http://csdmruk.cegedim.com/). Practices contributing to THIN are broadly representative of UK general practices in terms of patients’ age and sex, practice size and geographical distribution. GPs enter medical diagnoses and symptoms as Read codes, a hierarchical coding system used to record clinical information. This coding system maps onto ICD-10 codes including F40–F41 (from the Neurotic, stress-related and somatoform chapter) and in addition has a range of other possible codes which can be selected including terms such as ‘anxiety states’ and symptoms such as ‘anxiousness’. Procedures, prescriptions, health promotion activity and referrals to secondary care are also recorded. Recording of consultations and prescriptions is comparable to national consultation and prescription statistics [14]. Disease rates have been calculated from the data and compared to externally generated rates in validation studies [15].

### Measurements

Read code lists were compiled and used to identify GP recording of i) diagnoses of anxiety diagnoses (eg. chronic anxiety, generalised anxiety disorder, anxiety state), ii) anxiety symptoms (e.g. ‘anxiousness’), iii) mixed anxiety and depression, iv) panic attacks and v) panic disorder. Codes for phobias, Obsessive Compulsive Disorder and Post Traumatic Stress Disorder were not included.

A new episode was defined as an entry in the records with no previous entry of that problem recorded in the previous year. Participants could have more than one new episode within the study follow-up period, providing there was 12 months between episodes. We excluded data from the first 12 months following new registration with a THIN practice, as this may be retrospective recording of a past history rather than a true incident recording of a new episode of anxiety or panic [16]. Age was categorised into five age bands (16–24; 25–44; 45–64; 65–74; 75 and above). Deprivation was examined using quintiles of Townsend score using linkage to population census data for 2001 [17], and is a combined measure of owner-occupation, car ownership, overcrowding and unemployment [18] based on patient postcodes for approximately 150 households in that postal area.

For this study, data was only included for each participant where the practice had met pre-defined standards for acceptable data recording, including consistent recording of at least one medical record (e.g. a diagnostic entry), one additional health data record (e.g. a measurement or blood test result) and at least two prescriptions on average for the practice per patient per year and consistent reporting of mortality.

### Statistical Analysis

Analysis was conducted using Stata version 11. We calculated annual incidence rates by dividing the annual number of incident cases by the total person years at risk (PYAR) for each year. We determined uni-variable associations between year of diagnosis (1998 to 2008), gender, age group and quintiles of deprivation score, with incidence of recorded anxiety diagnosis, anxiety symptoms, mixed anxiety and depression and panic disorder/attacks. We conducted multi-variable Poisson regression to investigate the adjusted associations between incidences of each anxiety sub-group with year of diagnosis, gender, age group and deprivation, using robust standard errors to account for clustering within practices. The significance of variables in the Poisson regression modelling was assessed using Wald tests. We examined for interactions between age, gender, deprivation and period. There were statistically significant interactions between age and gender and age and deprivation. However, the sizes of these differences were relatively small, varied between anxiety sub-groups and were not clinically meaningful. We have presented the main results un-stratified, and the stratified incidence rate ratios by age-group, gender and deprivation are available in Table S1.

## Results

### Sample Characteristics

There were 3.7 million registered patients aged 16 years and over from January 1998 to March 2008, and 22 million patient years of follow-up time. There were 123,415 recorded new episodes of anxiety diagnoses; 109,672, new episodes of anxiety symptoms; 74,855 new episodes of mixed anxiety and depression, 41,774 new episodes of panic attacks and 14,602 new episodes of panic disorder. The median follow-up time was 5.5 years (IQR 3.0 to 8.5 years).

### GP Labelling of Anxiety and Panic in Health Care Records

The most frequently used Read codes for each category are given in Table 1. These were dominated by the use of certain codes, for example: ‘Anxiety state’ or ‘Anxiety state NOS’ was used in 110,661/123,415 (90%) of anxiety diagnoses episodes, ‘anxiousness’ or ‘anxiousness-symptom’ was used in 106,482/109,672 (97%) of anxiety symptom episodes, ‘anxiety with depression’ was used in 71,342/74,855 (95%) mixed anxiety and depression episodes. Codes for some specific ICD-10 categories were much less commonly used, for example ‘Generalised Anxiety Disorder’ was only documented in 6,816/123,415 (6%) of anxiety diagnoses) and ‘Mixed Anxiety and Depressive Disorder’ in 3,177/74,855 (4%) of those with mixed anxiety and depression codes).

**Table 1:** Frequently used Read codes by GPs to record anxiety diagnoses, symptoms, mixed anxiety and depression and panic

Description of Read code	FrequencyN (%)
**Anxiety diagnoses**	
Anxiety states	102294 (82.9)
Anxiety state NOS	8367 (6.8)
Generalised anxiety disorder	3683 (3.0)
[X]Generalized anxiety disorder	3133 (2.5)
Chronic anxiety	2120 (1.7)
**Anxiety symptoms**	
Anxiousness - symptom	71089 (64.8)
Anxiousness	35393 (32.3)
Tension - nervous	1112 (1.0)
‘Nerves’	1014 (0.9)
[D]Nervousness	455 (0.4)
**Mixed anxiety and depression**	
Anxiety with depression	71342 (95.3)
[X]Mixed anxiety and depressive disorder	3177 (4.2)
[X]Mild anxiety depression	336 (0.4)
**Panic attacks**	
Panic attack	33651 (80.6)
C/O - panic attack	7200 (17.2)
[X]Panic attack	624 (1.5)
[X]Panic state	299 (0.7)
**Panic disorder**	
Panic disorder	13538 (92.7)
[X]Panic disorder [episodic paroxysmal anxiety]	1064 (7.3)

[X]  =  These Read Codes were added to the original Read code dictionary, to ensure mapping to all ICD codes.

[D]  =  Working diagnosis.

C/O  =  Short-hand notation for Complaining of (commonly used by doctors to record symptoms in the UK).

### Trends Over Time

**Figure 1:**
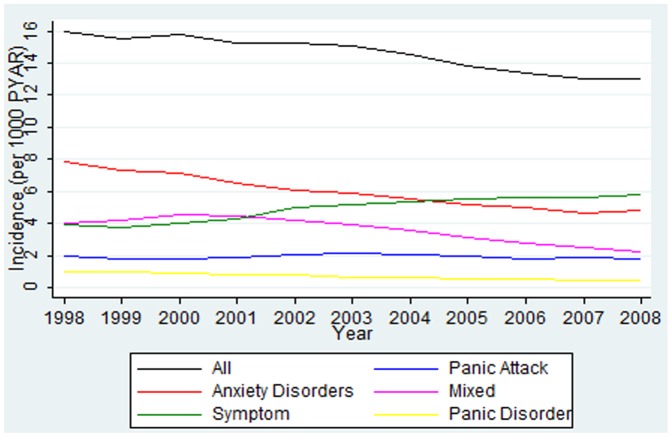
Time trends in Incidence of GP recorded Anxiety diagnoses, Mixed Anxiety and Depression, Anxiety Symptoms and Panic Attacks.

The combined incidence of anxiety diagnoses, symptoms, mixed anxiety and depression and panic fell slightly over time from 16/1000 Person Years at Risk (PYAR) in 1998 to 13/1000PYAR in 2008. Within this, time trends for anxiety sub-groups varied, with a reducing trend for anxiety diagnoses, mixed anxiety and depression and panic disorder, a rising trend for anxiety symptoms, and a more stable incidence of panic attacks over time (Figure 1).

### Incidence of Recorded Anxiety Diagnoses

The overall GP recorded incidence of anxiety diagnoses in the study period 1998–2008 was 5.7/1000PYAR (95%CI 5.7 to 5.8). The recorded incidence fell by a third from 7.9/1000PYAR (95%CI 7.7 to 8.1) in 1998 to 4.9/1000PYAR (95%CI 4.7 to 5.0) in 2008. (Table 2 and Figure 1) After adjustment for gender, age, deprivation and for clustering by general practice, the incidence rate ratio (IRR) was 0.64 (95%CI 0.56 to 0.73) when comparing 2008 to 1998. Recorded incidence in women was almost twice that of men and was highest in the middle aged to retirement group (Aged 45–64 years Adjusted IRR 1.73, in comparison to 16–24 years age group), falling again in older age groups. Recorded incidence of anxiety diagnoses also rose significantly with increasing deprivation (Adjusted IRR 1.65 in the most deprived compared to the least deprived groups).

**Table 2:** Incidence Rate Ratios for GP Recorded Anxiety Diagnoses.

Variable		No. of Events	PYAR	Incidence(95% CI)	Univariable* IRR(95% CI)	P-Value+	Multivariable* ∧ IRR(95% CI)	P-Value+
**Year**	1998	6941	879491	7.9 (7.7,8.1)	Baseline	<0.0001	Baseline	<0.0001
	1999	8328	1143029	7.3 (7.1,7.4)	0.92 (0.84,1.01)		0.93 (0.85,1.02)	
	2000	10730	1499653	7.2 (7.0,7.3)	0.91 (0.82,1.00)		0.92 (0.83,1.01)	
	2001	11521	1776784	6.5 (6.4,6.6)	0.82 (0.74,0.92)		0.84 (0.75,0.93)	
	2002	12518	2060714	6.1 (6.0,6.2)	0.77 (0.68,0.86)		0.79 (0.70,0.88)	
	2003	13586	2291800	5.9 (5.8,6.0)	0.75 (0.66,0.85)		0.77 (0.68,0.87)	
	2004	13495	2440041	5.5 (5.4,5.6)	0.70 (0.62,0.80)		0.72 (0.64,0.82)	
	2005	13488	2598986	5.2 (5.1,5.3)	0.66 (0.58,0.75)		0.68 (0.60,0.78)	
	2006	13523	2718023	5.0 (4.9,5.1)	0.63 (0.55,0.72)		0.65 (0.57,0.75)	
	2007	12963	2768774	4.7 (4.6,4.8)	0.59 (0.52,0.68)		0.62 (0.54,0.70)	
	2008	6322	1301973	4.9 (4.7,5.0)	0.62 (0.54,0.71)		0.64 (0.56,0.73)	
	TOTAL	123415	21479269	5.7 (5.7,5.8)				
**Gender**	Male	40628	10341390	3.9 (3.9,4.0)	Baseline	<0.0001	Baseline	<0.0001
	Female	82787	11137879	7.4 (7.4,7.5)	1.89 (1.85,1.94)		1.92 (1.87,1.97)	
**Age group**	16 to 24	10567	2739664	3.9 (3.8,3.9)	Baseline	<0.0001	Baseline	<0.0001
	25 to 44	46697	7870402	5.9 (5.9,6.0)	1.54 (1.48,1.60)		1.56 (1.50,1.62)	
	45 to 64	41242	63331832	6.5 (6.5,6.6)	1.69 (1.61,1.77)		1.73 (1.65,1.82)	
	65 to 74	12856	2235334	5.8 (5.7,5.9)	1.49 (1.40,1.59)		1.49 (1.40,1.58)	
	75+	12053	2302038	5.2 (5.1,5.3)	1.36 (1.26,1.47)		1.29 (1.20,1.40)	
**Deprivation**	Missing	4722	1801970	2.6 (2.5,2.7)	Excluded		Excluded	
(Least Deprived)	Q1	27311	5075427	5.4 (5.3,5.4)	Baseline	<0.0001	Baseline	<0.0001
	Q2	23452	4430050	5.3 (5.2,5.4)	0.98 (0.92,1.05)		0.99 (0.93,1.06)	
	Q3	23641	4153536	5.7 (5.6,5.8)	1.06 (0.98,1.14)		1.08 (1.00,1.16)	
	Q4	23807	3610922	6.6 (6.5,6.7)	1.23 (1.11,1.35)		1.26 (1.15,1.39)	
(Most Deprived)	Q5	20482	2407364	8.5 (8.4,8.6)	1.58 (1.37,1.82)		1.65 (1.44,1.90)	

* Standard Errors Adjusted For Practice;

+ P-value based on Wald test.

∧ Mutually adjusted for period, gender, age and deprivation and for clustering by practice using robust standard errors.

### Incidence of Recorded Anxiety Symptoms

The overall GP recorded incidence of anxiety symptoms in the study period was 5.1/1000PYAR (95%CI 5.07 to 5.13) (Table 3). The recorded incidence rose significantly by 50% over the decade, from 3.9/1000PYAR in 1998 to 5.8/1000PYAR in 2008 (Table 3 and Figure 1). Incidence in women was twice that of men and was highest in the middle aged to retirement group (45 to 64 years Adjusted IRR 1.70 compared to 16–24 years). Recorded incidence of anxiety symptoms also rose with increasing deprivation (Adjusted IRR 1.69 in the most deprived compared to the least deprived groups).

**Table 3:** Incidence Rate Ratios for Symptoms of Anxiety.

Variable		No of Events	PYAR	Incidence(95% CI)	Univariable* IRR(95% CI)	P-Value+	Multivariable* ∧ IRR(95% CI)	P-Value+
**Year**	1998	3456	882698	3.9 (3.8,4.0)	Baseline	<0.0001	Baseline	<0.0001
	1999	4351	1147001	3.8 (3.7,3.9)	0.97 (0.80,1.18)		0.97 (0.80,1.18)	
	2000	6036	1504479	4.0 (3.9,4.1)	1.03 (0.83,1.26)		1.04 (0.85,1.28)	
	2001	7621	1781717	4.3 (4.2,4.4)	1.09 (0.88,1.36)		1.11 (0.90,1.38)	
	2002	10371	2064140	5.0 (4.9,5.1)	1.28 (1.02,1.61)		1.30 (1.03,1.63)	
	2003	11920	2294092	5.2 (5.1,5.3)	1.33 (1.05,1.69)		1.34 (1.06,1.70)	
	2004	13135	2441491	5.4 (5.3,5.5)	1.37 (1.07,1.76)		1.39 (1.08,1.78)	
	2005	14284	2599104	5.5 (5.4,5.6)	1.40 (1.09,1.81)		1.42 (1.11,1.84)	
	2006	15342	2717026	5.6 (5.6,5.7)	1.44 (1.13,1.84)		1.47 (1.16,1.87)	
	2007	15651	2766365	5.7 (5.6,5.7)	1.45 (1.13,1.85)		1.47 (1.15,1.88)	
	2008	7505	1300640	5.8 (5.6,5.9)	1.47 (1.15,1.89)		1.51 (1.18,1.93)	
	TOTAL	109672	21498755	5.1 (5.1,5.1)				
**Gender**	Male	34943	10349131	3.4 (3.3,3.4)	Baseline	<0.0001	Baseline	<0.0001
	Female	74729	11149624	6.7 (6.7,6.8)	1.99 (1.93,2.04)		2.00 (1.95,2.05)	
**Age group**	16 to 24	9634	2740836	3.5 (3.4,3.6)	Baseline	<0.0001	Baseline	<0.0001
	25 to 44	39326	7879886	5.0 (4.9,5.0)	1.42 (1.36,1.48)		1.47 (1.40,1.53)	
	45 to 64	36308	6339300	5.7 (5.7,5.8)	1.63 (1.55,1.71)		1.70 (1.62,1.78)	
	65 to 74	12067	2236507	5.4 (5.3,5.5)	1.54 (1.43,1.64)		1.57 (1.47,1.68)	
	75+	12337	2302226	5.4 (5.3,5.5)	1.53 (1.40,1.66)		1.47 (1.35,1.59)	
**Deprivation**	Missing	3343	1803736	1.9 (1.8,1.9)	Excluded		Excluded	
(Least Deprived)	Q1	23461	5080576	4.6 (4.6,4.7)	Baseline	<0.0001	Baseline	<0.0001
	Q2	21155	4433446	4.8 (4.7,4.8)	1.03 (0.94,1.14)		1.03 (0.93,1.14)	
	Q3	22086	4156113	5.3 (5.2,5.4)	1.15 (1.03,1.28)		1.16 (1.04,1.29)	
	Q4	21589	3613983	6.0 (5.9,6.1)	1.29 (1.13,1.48)		1.32 (1.15,1.51)	
(Most Deprived)	Q5	18038	2410901	7.5 (7.4,7.6)	1.62 (1.37,1.92)		1.69 (1.43,1.99)	

* Standard Errors Adjusted For Practice;

+ P-value based on Wald test.

∧ Mutually adjusted for period, gender, age and deprivation and for clustering by practice using robust standard errors.

### Incidence of Mixed Anxiety and Depression

The incidence of recorded episodes of mixed anxiety and depression was 3.5/1000PYAR (95%CI 3.45 to 3.50) overall (Table 4). This recorded incidence nearly halved over the decade from 4.0/1000PYAR in 1998 to 2.2/1000PYAR in 2008. The incidence rate in women was twice that of men and was highest in both the 25–44 year (Adjusted IRR 1.80) and 45–64 year (Adjusted IRR 1.78) age groups and lowest in those over 75 years of age (Adjusted IRR 0.88) when compared with young adults aged 16–24years. Recorded incidence also rose with increasing deprivation, with those in the most deprived areas having almost twice the incidence rate of those in the least deprived areas.

**Table 4:** Incidence Rate Ratios for Mixed Anxiety and Depression.

Variable		No of Events	PYAR	Incidence(95% CI)	Univariable* IRR(95% CI)	P-value+	Multivariable* ∧ IRR(95% CI)	P-value+
**Year**	1998	3537	883306	4.0 (3.9,4.1)	Baseline	<0.0001	Baseline	<0.0001
	1999	4850	1146749	4.2 (4.1,4.4)	1.06 (0.93,1.20)		1.06 (0.93,1.21)	
	2000	6825	1503518	4.5 (4.4,4.6)	1.13 (0.97,1.32)		1.14 (0.98,1.33)	
	2001	7939	1780650	4.5 (4.4,4.6)	1.11 (0.96,1.29)		1.13 (0.97,1.31)	
	2002	8617	2064236	4.2 (4.1,4.3)	1.04 (0.89,1.23)		1.05 (0.89,1.24)	
	2003	9031	2295956	3.9 (3.9,4.0)	0.98 (0.83,1.17)		0.99 (0.83,1.18)	
	2004	8683	2444601	3.6 (3.5,3.6)	0.89 (0.74,1.06)		0.89 (0.75,1.06)	
	2005	8111	2604062	3.1 (3.1,3.2)	0.78 (0.65,0.93)		0.79 (0.66,0.94)	
	2006	7495	2723743	2.8 (2.7,2.8)	0.69 (0.56,0.84)		0.69 (0.57,0.84)	
	2007	6879	2774830	2.5 (2.4,2.5)	0.62 (0.49,0.78)		0.62 (0.50,0.77)	
	2008	2888	1305137	2.2 (2.1,2.3)	0.55 (0.44,0.69)		0.56 (0.45,0.68)	
	TOTAL	74855	21526788	3.5 (3.5,3.5)				
**Gender**	Male	24191	10357374	2.3 (2.3,2.4)	Baseline	<0.0001	Baseline	<0.0001
	Female	50664	11169414	4.5 (4.5,4.6)	1.94 (1.88,2.01)		2.01 (1.95,2.08)	
**Age group**	16 to 24	6389	2743476	2.3 (2.3,2.4)	Baseline	<0.0001	Baseline	<0.0001
	25 to 44	32558	7883564	4.1 (4.1,4.2)	1.77 (1.69,1.87)		1.80 (1.71,1.90)	
	45 to 64	25340	6347288	4.0 (3.9,4.0)	1.71 (1.60,1.83)		1.78 (1.67,1.89)	
	65 to 74	5643	2242792	2.5 (2.5,2.6)	1.08 (0.99,1.18)		1.09 (1.01,1.17)	
	75+	4925	2309669	2.1 (2.1,2.2)	0.92 (0.83,1.02)		0.88 (0.80,0.97)	
**Deprivation**	Missing	3023	1803736	1.7 (1.6,1.7)	Excluded		Excluded	
(Least Deprived)	Q1	15494	5086918	3.0 (3.0,3.1)	Baseline	<0.0001	Baseline	<0.0001
	Q2	13901	4439284	3.1 (3.1,3.2)	1.03 (0.92,1.14)		1.04 (0.94,1.16)	
	Q3	14492	4162459	3.5 (3.4,3.5)	1.14 (1.01,1.30)		1.17 (1.03,1.32)	
	Q4	15109	3619361	4.2 (4.1,4.2)	1.37 (1.17,1.60)		1.41 (1.21,1.65)	
(Most Deprived)	Q5	12836	2415030	5.3 (5.2,5.4)	1.75 (1.43,2.13)		1.82 (1.50,2.21)	

* Standard Errors Adjusted For Practice;

+ P-value based on Wald test.

∧ Mutually adjusted for period, gender, age and deprivation and for clustering by practice using robust standard errors.

### Incidence of Panic Attacks and Panic Disorder

The overall incidence of recorded episodes of panic attacks was 1.9/1000PYAR (95%CI 1.9 to 2.0) and panic disorder was 0.7/1000PYAR (95%CI 0.7 to 0.7) (Tables 5 and 6). The recorded incidence of panic attacks was stable over time, but the incidence of panic disorder reduced over time from 0.9/1000PYAR in 1998 to 0.5/1000PYAR in 2008. Both panic attacks and panic disorder episodes were more than twice as common in women compared to men and their incidence fell with increasing age, with those over 75 years having nearly half the recorded incidence of young people aged 16–24 years. Incidence of both panic attacks and panic also rose with increasing deprivation, with those in the most deprived areas having nearly twice the incidence rates of those in the least deprived areas.

**Table 5:** Incidence Rate Ratios for Panic Attacks.

Variable		No. of Events	PYAR	Incidence(95% CI)	Univariable* IRR(95% CI)	P-Value+	Multivariable* ∧ IRR(95% CI)	P-Value+
**Year**	1998	1740	884855	2.0 (1.9,2.1)	Baseline	<0.0001	Baseline	<0.0001
	1999	2068	1149351	1.8 (1.7,1.9)	0.91 (0.83,1.00)		0.92 (0.84,1.01)	
	2000	2739	1507574	1.8 (1.7,1.9)	0.92 (0.82,1.03)		0.93 (0.83,1.04)	
	2001	3423	1785764	1.9 (1.9,2.0)	0.97 (0.87,1.10)		0.99 (0.88,1.11)	
	2002	4201	2069896	2.0 (2.0,2.1)	1.03 (0.91,1.17)		1.05 (0.93,1.18)	
	2003	4915	2301418	2.1 (2.1,2.2)	1.09 (0.96,1.23)		1.10 (0.97,1.25)	
	2004	5077	2449652	2.1 (2.0,2.1)	1.05 (0.93,1.19)		1.07 (0.95,1.21)	
	2005	5090	2608566	2.0 (1.9,2.0)	0.99 (0.87,1.13)		1.01 (0.89,1.14)	
	2006	4993	2727744	1.8 (1.8,1.9)	0.93 (0.82,1.05)		0.95 (0.84,1.07)	
	2007	5145	2777964	1.9 (1.8,1.9)	0.94 (0.83,1.07)		0.96 (0.85,1.09)	
	2008	2383	1306350	1.8 (1.8,1.9)	0.93 (0.82,1.05)		0.94 (0.83,1.07)	
	TOTAL	41774	21569138	1.9 (1.9,2.0)				
**Gender**	Male	12108	10372503	1.2 (1.1,1.2)	Baseline	<0.0001	Baseline	<0.0001
	Female	29666	11196634	2.7 (2.6,2.7)	2.27 (2.20,2.34)		2.34 (2.27,2.41)	
**Age group**	16 to 24	5901	2744119	2.2 (2.1,2.2)	Baseline	<0.0001	Baseline	<0.0001
	25 to 44	17767	7901654	2.2 (2.2,2.3)	1.05 (1.00,1.09)		1.08 (1.03,1.13)	
	45 to 64	11967	6365161	1.9 (1.8,1.9)	0.87 (0.83,0.92)		0.92 (0.87,0.97)	
	65 to 74	3215	2246019	1.4 (1.4,1.5)	0.67 (0.63,0.71)		0.68 (0.64,0.72)	
	75+	2924	2312184	1.3 (1.2,1.3)	0.59 (0.55,0.63)		0.56 (0.52,0.60)	
**Deprivation**	Missing	1392	1805612	0.8 (0.7,0.8)	Excluded		Excluded	
(Least Deprived)	Q1	8187	5096334	1.6 (1.6,1.6)	Baseline	<0.0001	Baseline	<0.0001
	Q2	7529	4447572	1.7 (1.7,1.7)	1.05 (0.99,1.12)		1.06 (0.99,1.13)	
	Q3	8540	4170082	2.0 (2.0,2.1)	1.27 (1.20,1.35)		1.27 (1.20,1.35)	
	Q4	8884	3627286	2.4 (2.4,2.5)	1.52 (1.41,1.65)		1.51 (1.41,1.63)	
(Most Deprived)	Q5	7242	2422250	3.0 (2.9,3.1)	1.86 (1.70,2.03)		1.85 (1.69,2.02)	

* Standard Errors Adjusted For Practice;

+ P-value based on Wald test.

∧ Mutually adjusted for period, gender, age and deprivation and for clustering by practice using robust standard errors.

**Table 6:** Incidence Rate Ratios for Panic Disorder.

Variable		No. of Events	PYAR	Incidence(95% CI)	Univariable* IRR(95% CI)	P-Value+	Multivariable* ∧ IRR(95% CI)	P-Value+
**Year**	1998	839	885658	0.9 (0.9,1.0)	Baseline	<0.0001	Baseline	<0.0001
	1999	1165	1150276	1.0 (1.0,1.1)	1.07 (0.93,1.23)		1.09 (0.94,1.26)	
	2000	1375	1508653	0.9 (0.9,1.0)	0.96 (0.83,1.12)		0.98 (0.84,1.14)	
	2001	1489	1787435	0.8 (0.8,0.9)	0.88 (0.75,1.03)		0.91 (0.77,1.06)	
	2002	1662	2072163	0.8 (0.8,0.8)	0.85 (0.71,1.01)		0.88 (0.75,1.03)	
	2003	1563	2304423	0.7 (0.6,0.7)	0.72 (0.60,0.86)		0.74 (0.62,0.88)	
	2004	1505	2453276	0.6 (0.6,0.6)	0.65 (0.53,0.79)		0.68 (0.56,0.82)	
	2005	1510	2612172	0.6 (0.5,0.6)	0.61 (0.50,0.74)		0.64 (0.53,0.77)	
	2006	1462	2731408	0.5 (0.5,0.6)	0.57 (0.47,0.68)		0.59 (0.49,0.71)	
	2007	1382	2781746	0.5 (0.5,0.5)	0.52 (0.43,0.64)		0.56 (0.46,0.67)	
	2008	650	1308178	0.5 (0.5,0.5)	0.52 (0.43,0.64)		0.55 (0.45,0.67)	
	TOTAL	14602	21595391	0.7 (0.7,0.7)				
**Gender**	Male	4267	10379918	0.4 (0.4,0.4)	Baseline	<0.0001	Baseline	<0.0001
	Female	10335	11215472	0.9 (0.9,0.9)	2.24 (2.14,2.35)		2.34 (2.23,2.46)	
**Age group**	16 to 24	1768	2747904	0.6 (0.6,0.7)	Baseline	<0.0001	Baseline	<0.0001
	25 to 44	6527	7912587	0.8 (0.8,0.8)	1.28 (1.20,1.37)		1.29 (1.21,1.38)	
	45 to 64	4396	6372629	0.7 (0.7,0.7)	1.07 (1.00,1.15)		1.09 (1.01,1.17)	
	65 to 74	1097	2248082	0.5 (0.5,0.5)	0.76 (0.69,0.84)		0.75 (0.67,0.82)	
	75+	814	2314188	0.4 (0.3,0.4)	0.55 (0.48,0.62)		0.51 (0.45,0.57)	
**Deprivation**	Missing	789	1806106	0.4 (0.4,0.5)	Excluded		Excluded	
(Least Deprived)	Q1	2842	5101528	0.6 (0.5,0.6)	Baseline	<0.0001	Baseline	<0.0001
	Q2	2589	4452337	0.6 (0.6,0.6)	1.04 (0.93,1.17)		1.06 (0.95,1.18)	
	Q3	2819	4175626	0.7 (0.7,0.7)	1.21 (1.05,1.40)		1.22 (1.06,1.40)	
	Q4	2969	3633018	0.8 (0.8,0.8)	1.47 (1.26,1.71)		1.48 (1.28,1.73)	
(Most Deprived)	Q5	2594	2426774	1.1 (1.0,1.1)	1.92 (1.57,2.35)		1.94 (1.59,2.37)	

* Standard Errors Adjusted For Practice;

+ P-value based on Wald test.

∧ Mutually adjusted for period, gender, age and deprivation and for clustering by practice using robust standard errors.

## Discussion

### Key Findings

There was a trend showing a reduced incidence of anxiety diagnoses, panic disorder and mixed anxiety and depression being recorded in patient records, alongside a corresponding increase in the recorded incidence of anxiety symptoms over the decade 1998–2008. The incidence of panic attacks has been more stable over time. The recorded incidence of all five sub-groups of anxiety was approximately twice as high in women as men. For anxiety diagnoses, anxiety symptoms and mixed anxiety and depression the recorded incidence was highest in the middle aged to pre/early retirement group (45–64years). Panic disorder and panic attacks, however, were more commonly recorded in younger people (25–44years) and the incidence fell in later life (those aged 65 years or more). The recorded incidence of all sub-types (including panic) was increased in those living in more deprived areas.

These findings may be explained by changes in GPs’ recording behaviour, with an increased preference for recording symptoms over anxiety diagnoses in more recent years, rather than a true decrease in the incidence of anxiety diagnoses, panic disorder and mixed anxiety and depression. Over the past decade there has been increasing debate about the meaning of psychiatric disease categories, in particular for those with milder presentations common in primary care [19]. This may have influenced GPs and led to an increased reluctance to use more distinct diagnostic labels to record people’s distress over time. The GPs in our study demonstrated a preference for broader diagnostic labels such as ‘anxiety states’, in comparison to more specific ICD-10 diagnoses, such as Generalised Anxiety Disorder or Mixed Anxiety and Depressive Disorder. This may reflect uncertainties or lack of training in the criteria needed for these diagnoses, beliefs that the distinctions are not meaningful in primary care practice or will not impact on the management plan, or reluctance to use formal psychiatric diagnoses in patients’ records which may be perceived as stigmatising for patients.

### Comparison with Other Studies

There have been few previous epidemiological studies on anxiety disorders in primary care settings, in particular studies of incidence, with which to compare this work. Our study reports on GP recording of anxiety over a ten year period and, in order to be included, people would have needed to both seek help for their anxiety or consult with another problem, and to have their anxiety symptoms identified and considered sufficiently important for the GP to record them in their electronic health record. One other UK study has reported the incidence of GP recorded anxiety, although it did not differentiate between sub-types of anxiety and included both symptoms and diagnoses combined. It found a combined recorded incidence of anxiety of 9.7/1000PYAR [12] which is lower than our combined incidence of anxiety diagnoses, symptoms, mixed anxiety and depression, panic attacks and panic disorder of 14.4/1000PYAR over the period 1998–2008. This study also interrogated a primary care database, but had different selection criteria and anxiety sub-types, a younger age-range (10–79years) and was limited to 2002–4 [12]. We have not found any other studies of trends in GP recorded incidence of anxiety, but found similar trends of rising incidence of symptoms and falling incidence of diagnoses over time in the recording of depression by GPs in UK primary care [13]. Studies of time trends using a repeated community survey design have found differing trends, for example in Sweden prevalence rates of self-reported anxiety have increased in younger people but remained stable in older people [20] and prevalence of common mental disorder and symptoms of ‘worry’ have remained relatively stable over successive waves of the British National Survey of Psychiatric Morbidity 1993–2007 [21].

Our findings of an increased incidence of all types of anxiety reported in women are consistent with past community studies in screened populations [5], [9], [22], [23], and are therefore unlikely to reflect a bias of GPs recording more anxiety in women than men. Community-based studies have shown similar patterns as regards age and anxiety disorders, with an increased incidence of panic disorder in the age range of 20–54 years of age, and an increased incidence of Generalised Anxiety Disorder in the slightly older age group of 30–54 years [9]. However, in the previous study of GP recorded anxiety episodes where all diagnostic groups were combined, there was an increased incidence in the younger age band of 20–29 years [12]. It is not clear why the recorded incidence of anxiety symptoms and anxiety diagnoses is higher in the middle aged to pre/early retirement years, while panic and depression [13] appears to be more common in younger age groups. This may reflect either differences in the way GPs identify or choose to define psychological problems at different life stages or true differences in underlying incidence. Underlying differences in incidence across age-groups for different types of common mental disorder may be related to changing stressors and responses to these across the life-course.

Our incidence rates for GP recorded episodes of anxiety diagnoses were lower than incidence rates from screened populations in primary care waiting rooms, where the 6 month incidence of anxiety/panic was 5.5% [24]. We allowed for a 12 month gap between recorded incident episodes in our study, and this may have underestimated our incidence by excluding short-lived episodes that re-occurred within a year from onset. Our lower incidence rates may indicate that only a small proportion of people with anxiety and panic disorders seen in primary care have the problem identified and recorded in their health care record, which suggests either under detection or reluctance to disclose or document anxiety disorders in this setting. Previous work has suggested that few people with milder symptoms of anxiety, such as those with mixed anxiety and depressive disorder, seek help from their GP for these symptoms, although they are attending on a regular basis for other reasons [25]. Community incidence rates of anxiety disorders from screening using psychiatric diagnostic interviews in large population surveys are more similar to the incidence rates found in our study, although with different sub-groups included, for example varying from 11.1/1000PYAR including anxiety and mixed affective disorders [26] to 1.57% for anxiety disorders including panic, phobias and generalised anxiety [9]. In our study the incidence of GP recorded mixed anxiety and depression was low, and yet this was the largest group of common mental disorder identified in the UK National Psychiatric Morbidity survey, with a two-week prevalence as high as 9% [3], suggesting that GPs either greatly under-diagnose this group or code milder mixed presentations of anxiety and depression in other ways, such as using codes for depression alone. There is some evidence from studies of referrals to psychological therapies in the UK that GPs tend to under-record anxiety as a presenting problem in the presence of depression [27], [28].

### Strengths and Limitations

This is the first large nationally representative study which documents the trends in recording of anxiety diagnoses and symptoms in primary care over a 10 year period, including more than 360,000 incident episodes. Anxiety diagnoses recorded by GPs in the UK have been validated in previous work and show a high specificity for anxiety, where 121/135 (89.6%) of those with recorded diagnoses had confirmed diagnoses using questionnaires [12]. However, the sensitivity of GP recorded anxiety is unknown and may be much lower. There is also evidence from the USA for high levels of agreement between screening instruments (the PRIME-MD, Hamilton Anxiety Rating Scale A and the Panic Disorder Severity Scale), and primary care physician diagnoses for panic disorder and GAD, with agreement in 321/329 (98%) of participants [29]. In our study we included GAD within a broader ‘anxiety diagnoses’ group, as in the Read coding system codes for GAD are a subset of ‘anxiety states’ lower down in the hierarchical coding tree, and we were uncertain of the consistency with which they were separately identified. It is likely that, in addition to those with recorded anxiety, there are those with anxiety which was recognised and discussed with their GP, but not coded in their record. There is some previous evidence of under-detection of anxiety in primary care setting, which may be mis-diagnosed, for example when presenting as physical symptoms or in association with a depressive disorder. There is also likely to be a further population where the anxiety was not detected or help not actively sought [10], [11]. GPs may in some cases have chosen to record anxiety symptoms as non-coded free text alongside other symptoms or diagnoses, or have used other non-specific codes not included in this study, such as ‘stress-related problem’. It may be that only those with more severe symptoms of anxiety are recorded in GP records. In this study to limit our analysis of smaller sub-groups, we did not include evaluation of phobias, obsessive-compulsive disorder and post-traumatic stress disorder, and further work would be recommended to consider socio-demographic trends in incidence of these disorders in primary care. This study reflects the current recognition and recording practices by clinicians in UK primary care for anxiety diagnoses/symptoms, panic attacks/disorder and mixed anxiety and depression over time, and may not reflect the true incidence rates in this setting, which may be higher. More research is needed to clarify this. Our results are not generalisable to anxiety presenting to other settings, such as secondary care psychology or psychiatry.

### Implications

Recorded episodes of anxiety diagnoses, panic disorder and mixed anxiety and depression fell over time, whilst the recording of anxiety symptoms increased. This may reflect changes in the attitudes of GPs to recording psychiatric disorder or true changes in incidence over time.

Clinical databases are a rich potential source of data on mental health problems and psychiatric disorders, however researchers and policy makers should be aware that in the UK GPs use predominately non-specific or descriptive codes such as ‘anxiety state’ or ‘anxiousness’ in recording anxiety presentations and it is difficult to compare clinical practice using routinely collected data with findings from epidemiological surveys using standardised diagnostic instruments. Recorded incidence rates for anxiety are lower than in screened populations in this setting, and further work is needed to determine whether GPs are using other methods of recording, such as free text entries or coding presentations as depression when symptoms co-exist. We need to estimate the sensitivity of recorded anxiety diagnoses, and explore reasons why anxiety may be unrecognised in primary care, or acknowledged and discussed but not recorded.

## Supporting Information

Table S1 Recorded incidence by anxiety sub-group, stratified for interactions between age group with gender and age group with deprivation.
(DOCX)

## References

[1] Kessler RC, Berglund P, Demler O, Jin R, Merikangas KR, et al (2005) Lifetime prevalence and age of onset distributions of DSM-IV disorders in the National Comorbidity Survey Replication. Arch Gen Psychiatry 62: 93–602. 10.1001/archpsyc.62.6.593 15939837

[2] Somers JM, Goldner EM, Waraich P, Hsu L (2006) Prevalence and Incidence Studies of Anxiety Disorders: A Systematic Review of the Literature. Can J Psychiatry 51: 100–113. 16989109 10.1177/070674370605100206

[3] McManus S, Meltzer H, Brugha T, Bebbington P, Jenkins R (2009) Adult psychiatric morbidity in England, 2007: results of a household survey. London: National Centre for Social Research.

[4] King M, Nazareth I, Levy G, Walker C, Morris R, et al (2008) Prevalence of common mental disorders in general practice attendees across Europe Br J Psychiatry. 192: 362–367. 10.1192/bjp.bp.107.039966 18450661

[5] Stein MB, Roy-Byrne PP, Craske MG, Bystritsky A, Sullivan G, et al (2005) Functional impact and health utility of anxiety disorders in primary care outpatients. Med Care. 43: 1164–70. 10.1097/01.mlr.0000185750.18119.fd 16299426

[6] Kroenke K, Spitzer RL, Williams JBW, Monahan PO, Lowe B (2007) Anxiety Disorders in Primary Care: Prevalence, Impairment, Comorbidity, and Detection Ann Int Med. 146: 317–325. 10.7326/0003-4819-146-5-200703060-00004 17339617

[7] Greenberg PE, Sisitsky T, Kessler RC, Finkelstein SN, Berndt ER, et al (1999) The economic burden of anxiety disorders in the 1990s. J Clin Psychiatry 60: 427–435. 10453795 10.4088/jcp.v60n0702

[8] McCrone P, Dhanasiri S, Patel A, Knapp M, Lawton-Smith S (2008) Paying the price: the cost of mental health care in England to 2026. London: King’s Fund.

[9] Grant BF, Goldstein RB, Chou SP, Huang B, Stinson FS, et al (2009) Sociodemographic and psychopathologic predictors of first incidence of DSM-IV substance use, mood and anxiety disorders: results from the Wave 2 National Epidemiologic Survey on Alcohol and Related Conditions. Mol Psychiatry 14: 1051–1066. 18427559 10.1038/mp.2008.41 PMC2766434

[10] Wittchen HU, Kessler RC, Beesdo K, Krause P, Hofler M, et al (2002) Generalized anxiety and depression in primary care: prevalence, recognition and management. J Clin Psychiatry. 63 Suppl 824–34. 12044105

[11] Jameson JP, Blank MB (2010) Diagnosis and treatment of depression and anxiety in Rural and Non-rural Primary Care: National Survey Results. Psychiatric Services 61: 624–627. 20513688 10.1176/ps.2010.61.6.624

[12] Martin-Merino E, Ruigomez A, Wallander MA, Johansson S, Garcia-Rodriguez LA (2010) Prevalence, incidence, morbidity and treatment patterns in a cohort of patients diagnosed with anxiety in UK primary care. Fam Pract 27: 9–16 doi: 10.1093/fampra/cmp071. 19884124 10.1093/fampra/cmp071

[13] Rait G, Walters K, Griffin M, Buszewicz M, Petersen I, et al (2009) Recent trends in the incidence of recorded depression and depressive symptoms in primary care. Br J Psychiatry 195: 520–524 doi: 10.1192/bjp.bp.108.058636. 19949202 10.1192/bjp.bp.108.058636

[14] Bourke A, Dattani H, Robinson M (2004) Feasibility study and methodology to create a quality-evaluated database of primary care data. Inform Prim Care 12: 171–7. 15606990 10.14236/jhi.v12i3.124

[15] Lewis JD, Schinnar R, Bilker WB, Wang X, Strom BL (2007) Validation studies of the health improvement network (THIN) database for pharmacoepidemiology research. Pharmacoepidemiol Drug Saf 16: 393–401. 17066486 10.1002/pds.1335

[16] Lewis JD, Bilker WB, Weinstein RB, Strom BL (2005) The relationship between time since registration and measured incidence rates in the General Practice Research Database. Pharmacoepidemiol Drug Saf 14: 443–51. 15898131 10.1002/pds.1115

[17] Office of National Statistics (2001) Census 2001. Available: http://www.ons.gov.uk/ons/rel/census/census-2001-key-statistics/health-areas-in-england-and-wales/index.html. Accessed 2012 Jun 22..

[18] Townsend P, Phillimore P, Beattie A (1986) Inequalities in health in the northern region. Newcastle upon Tyne: Northern Regional Health Authority and University of Bristol.

[19] Hickie I, Parker G (2007) Is depression overdiagnosed? BMJ 335: 328–9. 17703040 10.1136/bmj.39268.475799.AD PMC1949440

[20] Kosidou K, Magnusson C, Mittendorfer-Rutz E, Hallqvist J, Gumpert CH, et al (2009) Recent time trends in levels of self-reported anxiety, mental health service use and suicidal behaviour in Stockholm. Acta Psychiatr Scand. doi: 10.1111/j.1600–0447.2009.01487.x. 10.1111/j.1600-0447.2009.01487.x 19824989

[21] Spiers N, Bebbington P, McManus S, Brugha TS, Jenkins R, et al (2011) Age and birth cohort differences in the prevalence of common mental disorder in England: National Psychiatric Morbidity Surveys 1993–2007 Br J Psychiatry. 198: 479–484 doi: 10.1192/bjp.bp.110.084269. 10.1192/bjp.bp.110.084269 21628710

[22] Eaton WW, Kramer M, Anthony JC, Dryman A, Shapiro S, et al (1989) The incidence of specific DIS/DSM-III mental disorders: data from the NIMH Epidemiologic Catchment Area program. Acta Psychiatr Scand 79: 163–178. 2784251 10.1111/j.1600-0447.1989.tb08584.x

[23] Bijl RV, De Graaf R, Ravelli A, Smit F, Vollebergh WAM (2002) Gender and age-specific first incidence of DSM-III-R psychiatric disorders in the general population: results from the Netherlands Mental Health Survey and Incidence Study (NEMESIS). Soc Psychiatry Psychiatr Epidemiol 37: 372–379. 12195544 10.1007/s00127-002-0566-3

[24] King M, Bottomley C, Bellón-Saameno, Torres-Gonzalez F, Švab I, et al (2011) An international risk prediction algorithm for the onset of generalized anxiety and panic syndromes in general practice attendees: predictA. Psychol Med. doi: 10.1017/S0033291710002400. 10.1017/S0033291710002400 21208520

[25] Walters K, Buszewicz M, Weich S, King M (2011) Outcomes of mixed anxiety and depressive disorder in primary care: a cohort study. Br J Psychiatry 198: 1–7 doi: 10.1192/bjp.bp.110.085092. 21628709 10.1192/bjp.bp.110.085092

[26] Murphy JM, Olivier DC, Monson RR, Sobol AM, Leighton AH (1988) Incidence of Depression and Anxiety: the Stirling County Study. Am J Pub Health 78: 534–540. 3258479 10.2105/ajph.78.5.534 PMC1349333

[27] Clark DM, Layard R, Smithies R, Richards DA, Suckling R, et al (2009) Improving access to psychological therapy: initial evaluation of two UK demonstration sites. Behaviour Research & Therapy 47: 910–920. 19647230 10.1016/j.brat.2009.07.010 PMC3111658

[28] Richards DA, Suckling R (2009) Improving access to psychological therapies: Phase IV prospective cohort study. Br J Clin Psychology 48: 377–396 doi: 10.1348/014466509X405178. 10.1348/014466509X405178 19208291

[29] Rollman BL, Belnap BH, Hum B, Mazumdar S, Zhu F, et al (2005) Symptomatic Severity of Prime-MD Diagnosed Episodes of Panic and Generalized Anxiety Disorder in Primary Care. J Gen Intern Med 20: 623–628. 16050857 10.1111/j.1525-1497.2005.0120.x PMC1490149

