# Supplementary material for: Recent Trends in the Incidence of Anxiety Diagnoses and Symptoms in Primary Care
Source: PLoS One. 2012 Aug 3;7(8):e41670. doi: 10.1371/journal.pone.0041670 (PMC3411689; doi:10.1371/journal.pone.0041670)
Supplement: Table S1 — Recorded incidence by anxiety sub-group, stratified for interactions between age group with gender and age group with deprivation. (DOCX) [file pone.0041670.s001.docx]

**Supporting Information Table 1: Recorded incidence by anxiety sub-group, stratified for interactions between age group with gender and age group with deprivation**

| **Variable** | | Age group | | | | |
| --- | --- | --- | --- | --- | --- | --- |
|  | | 16 to 24 yrs | 25 to 44 yrs | 45 to 64 yrs | 65 to 74 yrs | 75+ yrs |
|  | | Incidence Rate Ratio  (95% CI) | Incidence Rate Ratio  (95% CI) | Incidence Rate Ratio  (95% CI) | Incidence Rate Ratio  (95% CI) | Incidence Rate Ratio  (95% CI) |
| **Anxiety diagnoses** | |  |  |  |  |  |
| Gender | Male | Baseline | Baseline | Baseline | Baseline | Baseline |
|  | Female | 1.68(1.60,1.76) | 1.82(1.76,1.88) | 1.98(1.92,2.04) | 2.25(2.15,2.37) | 2.12(2.01,2.24) |
| Deprivation | (least)Q1 | Baseline | Baseline | Baseline | Baseline | Baseline |
|  | Q2 | 0.95(0.86,1.05) | 0.98(0.92,1.05) | 1.03(0.96,1.11) | 0.94(0.86,1.02) | 0.95(0.85,1.06) |
|  | Q3 | 1.08(0.97,1.19) | 1.09(1.01,1.18) | 1.09(1.00,1.18) | 0.99(0.89,1.09) | 1.060.95,1.19) |
|  | Q4 | 1.29(1.14,1.46) | 1.28(1.17,1.42) | 1.30(1.18,1.43) | 1.11(0.98,1.26) | 1.16(1.00,1.33) |
|  | (most)Q5 | 1.57(1.29,1.93) | 1.71(1.48,1.98) | 1.74(1.52,2.00) | 1.42(1.22,1.65) | 1.43(1.22,1.68) |
| **Anxiety symptoms** | |  |  |  |  |  |
| Gender | Male | Baseline | Baseline | Baseline | Baseline | Baseline |
|  | Female | 1.72(1.63,1.82) | 1.96(1.90,2.03) | 2.05(1.98,2.12) | 2.25(2.16,2.35) | 1.98(1.89,2.08) |
| Deprivation | (least)Q1 | Baseline | Baseline | Baseline | Baseline | Baseline |
|  | Q2 | 1.02(0.89,1.16) | 1.04(0.93,1.15) | 1.06(0.96,1.18) | 0.95(0.84,1.08) | 1.00(0.88,1.14) |
|  | Q3 | 1.09(0.96,1.24) | 1.19(1.05,1.33) | 1.17(1.05,1.31) | 1.13(0.99,1.29) | 1.13(1.00,1.29) |
|  | Q4 | 1.33(1.14,1.53) | 1.37(1.19,1.59) | 1.32(1.15,1.52) | 1.24(1.04,1.47) | 1.18(0.99,1.41) |
|  | (most)Q5 | 1.65(1.37,1.99) | 1.73(1.44,2.08) | 1.78(1.50,2.12) | 1.54(1.28,1.84) | 1.46(1.24,1.73) |
| **Mixed anxiety and depression** | |  |  |  |  |  |
| Gender | Male | Baseline | Baseline | Baseline | Baseline | Baseline |
|  | Female | 2.13(2.01,2.25) | 2.10(2.01,2.19) | 1.86(1.78,1.96) | 2.21(2.05,2.37) | 1.94(1.79,2.11) |
| Deprivation | (least)Q1 | Baseline | Baseline | Baseline | Baseline | Baseline |
|  | Q2 | 1.05(0.89,1.23) | 1.07(0.95,1.21) | 1.04(0.93,1.1) | 0.98(0.86,1.11) | 1.00(0.88,1.14) |
|  | Q3 | 1.30(1.10,1.54) | 1.17(1.02,1.56) | 1.17(1.02,1.34) | 1.10(0.94,1.30) | 1.06(0.90,1.25) |
|  | Q4 | 1.71(1.43,2.06) | 1.46(1.24,1.72) | 1.37(1.17,1.60) | 1.29(1.08,1.55) | 1.14(0.94,1.39) |
|  | (most)Q5 | 2.08(1.61,2.70) | 1.89(1.54,2.31) | 1.86(1.52,2.28) | 1.50(1.21,1.87) | 1.33(1.08,1.64) |
| **Panic disorder** | |  |  |  |  |  |
| Gender | Male | Baseline | Baseline | Baseline | Baseline | Baseline |
|  | Female | 2.43(2.27,2.60) | 2.44(2.35,2.55) | 2.33(2.22,2.44) | 2.11(1.95,2.30) | 1.86(1.69,2.04) |
| Deprivation | (least)Q1 | Baseline | Baseline | Baseline | Baseline | Baseline |
|  | Q2 | 1.02(0.90,1.14) | 1.07(0.99,1.16) | 1.07(0.98,1.16) | 1.05(0.92,1.19) | 1.00(0.87,1.16) |
|  | Q3 | 1.18(1.07,1.30) | 1.30(1.21,1.39) | 1.30(1.20,1.41) | 1.19(1.05,1.34) | 1.20(1.05,1.37) |
|  | Q4 | 1.37(1.24,1.52) | 1.55(1.43,1.68) | 1.60(1.46,1.76) | 1.48(1.27,1.72) | 1.30(1.10,1.55) |
|  | (most)Q5 | 1.53(1.33,1.77) | 1.92(1.74,2.13) | 2.04(1.84,2.26) | 1.74(1.50,2.02) | 1.60(1.38,1.86) |
| **Panic attacks** | |  |  |  |  |  |
| Gender | Male | Baseline | Baseline | Baseline | Baseline | Baseline |
|  | Female | 2.23(1.98,2.51) | 2.40(2.24,2.56) | 2.39(2.22,2.59) | 2.13(1.85,2.44) | 2.28(1.91,2.73) |
| Deprivation | (least)Q1 | Baseline | Baseline | Baseline | Baseline | Baseline |
|  | Q2 | 0.95(0.79,1.15) | 1.07(0.95,1.20) | 1.07(0.94,1.23) | 1.03(0.82,1.28) | 1.15(0.87,1.50) |
|  | Q3 | 1.06(0.87,1.30) | 1.34(1.16,1.56) | 1.22(1.03,1.44) | 0.89(0.70,1.12) | 1.21(0.90,1.61) |
|  | Q4 | 1.29(1.06,1.57) | 1.63(1.37,1.94) | 1.39(1.16,1.67) | 1.29(1.04,1.59) | 1.55(1.22,1.98) |
|  | (most)Q5 | 1.50(1.17,1.92) | 2.15(1.74,2.64) | 2.05(1.65,2.55) | 1.51(1.13,2.03) | 1.70(1.22,2.37) |
